# Expression and activity of the TLR4/NF-κB signaling pathway in mouse intestine following administration of a short-term high-fat diet

**DOI:** 10.3892/etm.2013.1214

**Published:** 2013-07-10

**Authors:** NING WANG, HUGUO WANG, HUA YAO, QIN WEI, XIN-MIN MAO, TAO JIANG, JING XIANG, NA DILA

**Affiliations:** 1 Department of No. 1 Cadre Wards Medicine, First Affiliated Hospital of Xinjiang Medical University, Urumqi, Xinjiang 830011, P.R. China; 2 Department of Digestive and Vascular Surgery, First Affiliated Hospital of Xinjiang Medical University, Urumqi, Xinjiang 830011, P.R. China; 3 Department of Metabolic Disease, First Affiliated Hospital of Xinjiang Medical University, Urumqi, Xinjiang 830011, P.R. China; 4 Department of Biomedical Sciences, First Affiliated Hospital of Xinjiang Medical University, Urumqi, Xinjiang 830011, P.R. China; 5 Experimental Teaching Demonstration Centre, Pharmaceutical College of Xinjiang Medical University, Urumqi, Xinjiang 830011, P.R. China; 6 Department of Endocrinology, Xinjiang Petroleum Administration Bureau General Hospital, Urumqi, Xinjiang 830011, P.R. China

**Keywords:** Toll-like receptor 4/NF-κB, short-term high-fat diet, intestines, local inflammatory response

## Abstract

Insulin resistance in obesity is associated with chronic systemic low-grade inflammation. Although it has been shown that Toll-like receptor 4 (TLR4) in the liver, muscle and adipose tissue plays an important role in obesity-associated inflammation and insulin resistance, the effect of TLR4 activation in the intestine has not been investigated. The aim of this study was to explore the activation of the mouse intestinal TLR4/NF-κB signaling pathway following the administration of a short-term high-fat diet, as well as the function of the signaling pathway in the local enteric inflammatory response. The effect of the high-fat diet on TLR4 activation, NF-κB and phosphorylated IκB (PIκB) activity, and tumor necrosis factor (TNF)-α and IL-6 expression in the intestinal tissues of diet-induced obese C57BL/6 mice was investigated. The results demonstrated that the high-fat diet induced TLR4 mRNA and protein expression in intestinal tissues. TLR4/NF-κB signaling pathway activation gradually increased as the number of days of high-fat diet administration increased, and peaked on day 7. Additionally, activation of the signaling pathway reduced PIκB expression levels and increased TNF-α and IL-6 expression levels in intestinal tissues. Our results demonstrated that a short-term high-fat diet induces activation of the TLR4/NF-κB signaling pathway in intestinal tissues, which causes local intestinal low-grade inflammation. These data improve our understanding of the molecular events involved in intestinal low-grade inflammation, which may be the triggering factor for chronic systemic low-grade inflammation.

## Introduction

A great deal of attention has been paid to the theory of inflammation and certain researchers consider that diabetes, obesity and atherosclerosis represent a low-grade, chronic inflammatory condition. Pickup (1) hypothesized that stimulation from dietary surplus and other environmental factors results in the activation of a specific cell population of the innate immune system. Certain sentinel cells, including macrophages and fat cells, secrete tumor necrosis factor (TNF)-α, IL-6 and other inflammatory agents that cause a low-grade inflammatory condition and accordingly trigger insulin resistance, diabetes and related diseases. Toll-like receptor 4 (TLR4) is one of the receptors that is able to identify pathogenic microorganisms in a natural immune system, bind the specific ligand and produce corresponding inflammation following identification. One study identified that TLR4 is related to whole-body, low-grade chronic inflammatory diseases, including those mentioned above (2).

Previous studies have demonstrated that TLR4 may be a central link between insulin resistance, inflammation and obesity, and that a point mutation in TLR4, which inactivates the receptor, prevents the diet-induced obesity (DIO) activation of IκB kinase (IKKβ) and c-Jun NH2-terminal kinase (JNK), and inhibits insulin resistance, suggesting that TLR4 is a key modulator in the cross-talk between inflammatory and metabolic pathways (3–8).

TLR4 exogenous ligands include lipopolysaccharides (LPS), endogenous ligands, including free fatty acids (FFA) (9), high-molecular-weight sugar (10) and the surface of fungi polysaccharides (10). Myenteric neurons mediate LPS recognition via TLR4, resulting in neuronal cell death (11). Continuous FFA stimulation activates TLR4 signaling pathways; however, when TLR4 is blocked or knocked out, FFA stimulation is also blocked (12). In addition, triglycerides (TG) also activate TLR4/NF-κB and increase the expression of inflammatory cytokines. One study demonstrated that acute and chronic, physical exercise in DIO rats induces significant suppression in the TLR4 signaling pathway in the liver, muscle and adipose tissue, reduces LPS serum levels and improves insulin signaling and sensitivity (13). Therefore, low-grade inflammation triggered by a high-fat diet is closely associated with TLR4 expression. The main focal point of current research is TLR4 receptors in insulin-sensitive organs and tissues, including fat, liver, pancreas and skeletal muscle; however, studies concerning intestinal TLR4 have not yet been reported.

The intestines are not only primary immune organs, but they also have the most extensive surface area compared with other immune organs. The TLR4 receptors, which are distributed on the intestinal surface, recognize enteric pathogen-associated molecular patterns (PAMPs) and activate NF-κB. Then, signals incorporated into the corresponding promoter and enhancer DNA binding sites in the cytokine gene regulation area initiate gene transcription and expression (14). Induced cytokines further activate immunocytes or cytokine receptors, expand the immune reaction, cause excessive release of inflammatory mediators and produce a cascade reaction of inflammatory factors. As a sensor of endogenous lipid and fatty acids, TLR4 regulates metabolism and the immune system (15).

On the basis of data from previous studies, we hypothesize that intestinal TLR4, activated by components of the high-fat diet, may become a key trigger for intestinal low-grade inflammation. Thus, the present study aimed to analyze the effect of mouse intestinal TLR4/NF-κB signaling pathway activation caused by a short-term, high-fat diet, as well as the function of the signaling pathway in the local enteric inflammatory response.

## Materials and methods

### Animals

Adult male C57BL/6 mice (n=60) aged 6 weeks, weighing 18±2 g, were purchased from Xinjiang Medical University Animal Experimental Center (Urumqi, China). All animals were housed and used in accordance with the Chinese Regulations for Animal Care. This study was performed in strict accordance with the recommendations in the Guide for the Care and Use of Laboratory Animals of the National Institutes of Health. The animal use protocol was reviewed and approved by the Institutional Animal Care and Use Committee (IACUC) of the First Affiliated Hospital of Xinjiang Medical University. All experiments were conducted with the approval of the animal ethics committees of Xinjiang Medical University. The mice were randomly divided into six groups and each group of ten mice was fed with a high-fat diet for 0, 1, 3, 5, 7 and 9 days, respectively. The formula of the high-fat diet was added to common fodder as follows: 10% sucrose, 28% maltodextrin, 0.12% choline chloride, 10% lard, 27.5% casein, 0.24% methionine and 0.1% sodium chloride.

### Hematoxylin and eosin (H&E) staining

The mice were sacrificed by decapitation after 6 h of fasting in accordance with experimental requirements. The intestines were removed, absterged with physiological saline solution and then stored at −80°C until analysis.

A 1 cm length of intestinal tissue from each sample was obtained. A microscope slide with rehydrated tissue sections was fixed in alcohol. The slide was immersed for 30 sec with agitation by hand in H_2_O. The slide was dipped into a Coplin jar containing Mayer’s hematoxylin and agitated for 30 sec. The slide was rinsed with H_2_O for 1 min. The slide was stained with 1% eosin Y solution for 10–30 sec with agitation. The sections were dehydrated with two changes of 95% alcohol and two changes of 100% alcohol for 30 sec each. The alcohol was removed with two changes of xylene. One or two drops of mounting medium was added and the section was covered with a coverslip. The intestinal mucosa was observed by light microscopy using the single-blind method.

### Immunohistochemistry

Intestinal tissue was fixed with paraformalin (40 g/l), embedded in paraffin, sectioned using a microtome (4 μm), deparaffinized by xylene, dehydrated with a graded alcohol series, blocked with 10% goat serum for 30 min at room temperature in order to block nonspecific binding and repaired. TNF-α and IL-6 were detected using rabbit anti-TNF-α and anti-IL-6 polyclonal antibodies (Abs), followed by application of horseradish peroxidase (HRP)-labeled goat secondary Abs. The presence of brown staining in the cytoplasm and/or nuclei indicated that cells were positive for TNF-α or IL-6. Each sample was randomly assessed with five dyes at high magnification (x400). Positive cells were graded and scored according to a coloring scale: no coloring (−, 0 points), coloring area <25% (+, 1 point), coloring area 25–50% (++, 2 points) and coloring area >50% (+++, 3 points).

### Western blot analysis

Briefly, holoprotein extracts (70 mg) of the mouse intestinal tissue in each sample were electrophoresed on a 10% sodium dodecyl sulfate (SDS)-polyacrylamide gel and transferred to polyvinylidene fluoride (PVDF) membranes. Activated TLR4, NF-κB and phosphorylated IκB (PIκB) were detected using rabbit Abs. Membranes were blocked and incubated with appropriate Abs at 4°C overnight, then imaged by conjugation with a HRP-linked secondary antibody and enhanced chemiluminescence (ECL) detection reagent. All experiments were performed in triplicate with similar results. All protein expression was divided by the amount of β-actin of individual samples as analyzed by image software. The blots were analyzed by densitometry.

### Real-time polymerase chain reaction (PCR)

The mRNA expression was analyzed using reverse transcription (RT)-PCR. Total RNA from mouse intestines was extracted using TRIzol reagent (Invitrogen Life Technologies, Carlsbad, CA, USA). Each RNA sample (5 μg) was diluted and reverse-transcribed into complementary DNA (cDNA), to provide transcripts (3 μg) for amplification. The primer sequences for amplification of the cDNA were as follows: TLR4, forward: 5′-CACTGTTCTTCTCCTGCCTGAC-3′ and reverse, 5′-TGG TTGAAGAAGGAATGTCATC-3′); NF-κB, forward: 5′-CCT CTGGCGAATGGCTTTAC-3′ and reverse: 5′-GCTATGGAT ACTGCGGTCTGG-3′; β-actin, forward: 5′-CACGATGGA GGGGCCGGACTCATC-3′ and reverse: 5′-TAAAGACCTCTA TGCCAACACAGT-3′). The PCR conditions were as follows: i) 95°C for 2 min for one cycle; ii) 95°C for 45 sec; iii) 54°C for 45 sec; iv) 72°C for 1 min (modified for each primer set); v) steps ii, iii and iv were repeated for 30 cycles for TLR4 mRNA, 35 cycles for NF-κB mRNA and 27 cycles for β-actin mRNA; vi) 72°C 5 min for one cycle. The identification of the PCR fragments was confirmed by size following electrophoretic migration on ethidium bromide (0.5 mg/l)-stained agarose gels and imaging. The amount of the PCR products was imaged and expressed as optical density. The target cDNA present in each sample was corrected for the respective β-actin values.

### Statistical analysis

Data are presented as mean ± standard deviation (SD). Student t-tests were performed to determine the statistical significance of protein and mRNA expression levels among the different groups. Enumeration data was analyzed by a rank sum test. P<0.05 was considered to indicate a statistically significant difference.

## Results

### H&E staining

**Figure 1:**
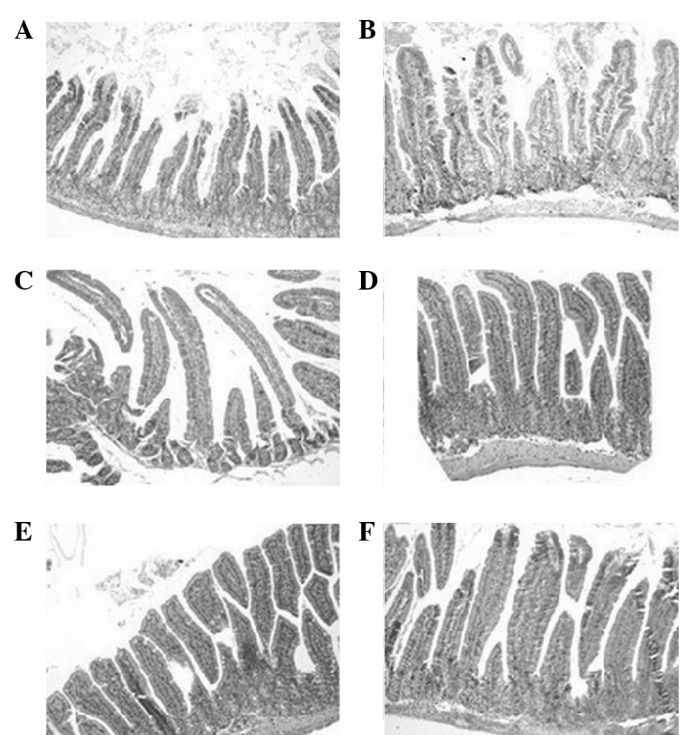
Mouse intestinal tissue with hematoxylin and eosin (H&E) staining. On days 0–9, the intestinal tissue was complete, with no neutrophilic granulocyte infiltration. On days 0–5, there were no macrophages in the intestinal tissue; however, a few macrophages were scattered and distributed on days 7 and 9, with no differences between the two groups. (A) Day 0; (B) day 1; (C) day 3; (D) day 5; (E) day 7; (F) day 9. Magnification, ×40.

By macroscopic observation, there were no clear changes and no hyperemia or hydrops in the enteric cavity. By light microscopic observation, there was no ulceration or interruption of the intestinal mucosa and no mass neutrophilic granulocyte infiltration. Diffused macrophage distribution was observed on days 7 and 9 (Fig. 1).

### Immunohistochemistry

**Figure 2:**
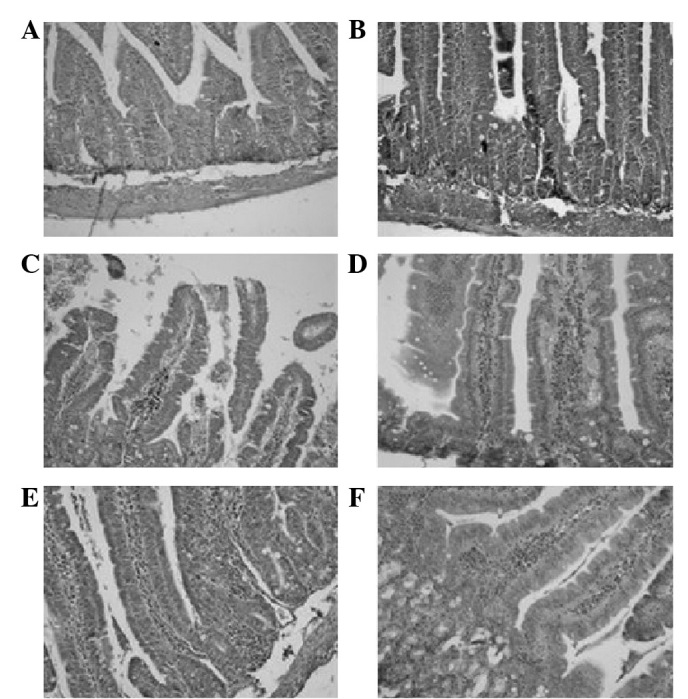
Mouse intestinal tissue with immunohistochemical staining of tumor necrosis factor (TNF)-α. On day 0 of high-fat diet stimulation, there were no TNF-α-positive cells. However, there were TNF-α-positive cells during the following days; a changing trend was observed with time. On day 1, there were a few cells expressing TNF-α that were continuously distributed in the intestinal epithelium. On day 3, the TNF-α-positive cells in the intestinal epithelium markedly deepened in color, while a few cells began to distribute in the lamina propria mucosa. On day 5, the TNF-α-positive cells in the lamina propria mucosa began to darken in color, increase in number and there was a clustering trend in the distribution. On day 7, the TNF-α-positive cells in the lamina propria mucosa were distributed in clusters; however, few were distributed and scattered in the submucosa. On day 9, the TNF-α-positive cells were distributed in clusters and dispersed in the lamina propria mucosa and submucosa. (A) Day 0; (B) day 1; (C) day 3; (D) day 5; (E) day 7; (F) day 9. Magnification, ×40.

**Figure 3:**
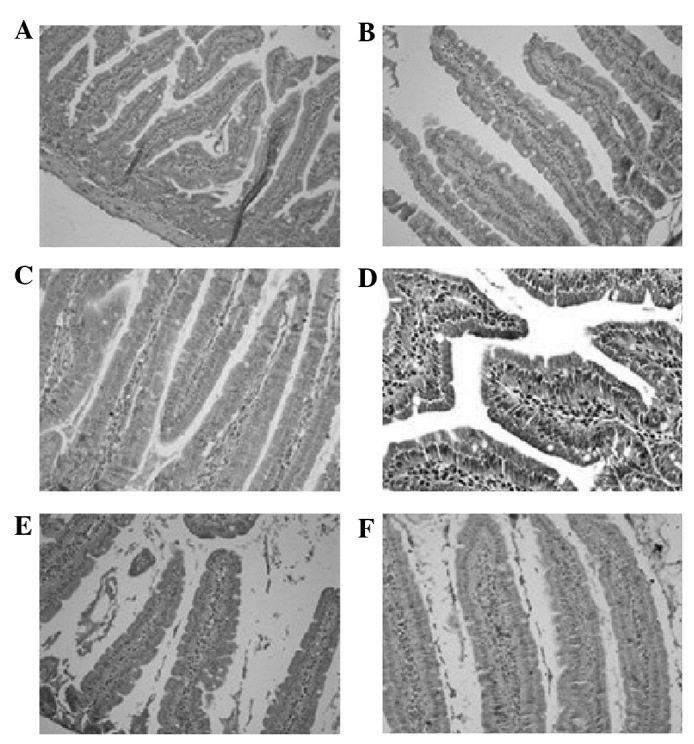
Mouse intestinal IL-6 immunohistochemical staining. On day 0 of high-fat diet stimulation, there were no IL-6-positive cells in the intestines. However, there were IL-6-positive cells during the following days, which gradually changed over time on days 1–9. On day 1, there were a few cells weakly expressing IL-6, which were sporadically distributed in the intestinal epithelium. On day 3, the IL-6-positive cells in the intestinal epithelium were continuously distributed and the color markedly deepened. There were a few pale colored IL-6-positive cells in the lamina propria mucosa. On day 5, the IL-6-positive cells in the lamina propria mucosa darkened in color; however, no changes in quantity or range occurred. On day 7, the amount of IL-6-positive cells in the lamina propria mucosa increased markedly and demonstrated a clustering trend in distribution. On day 9, there were IL-6-positive cells distributed in clusters, dispersed in the lamina propria mucosa but not in the submucosa. (A) Day 0; (B) day 1; (C) day 3; (D) day 5; (E) day 7; (F) day 9. Magnification, ×40.

All the groups expressed TNF-α and IL-6, with the exception of the day 0 group. The presence of brown staining in the cytoplasm and/or nuclei indicated that cells were positive for TNF-α and IL-6, which were mainly expressed in the nuclei. We observed that the quantity and distribution of TNF-α-positive cells presented dynamic changes with sustained stimulation by a high-fat diet. The expression gradually strengthened and demonstrated cluster distribution in the epithelial mucosa, lamina propria mucosa and submucosa (Table I and Fig. 2). The expression of IL-6-positive cells increased gradually in the epithelial mucosa and lamina propria mucosa, and diffused distribution demonstrated a clustering trend (Table II and Fig. 3).

### Western blot analysis

**Figure 4:**
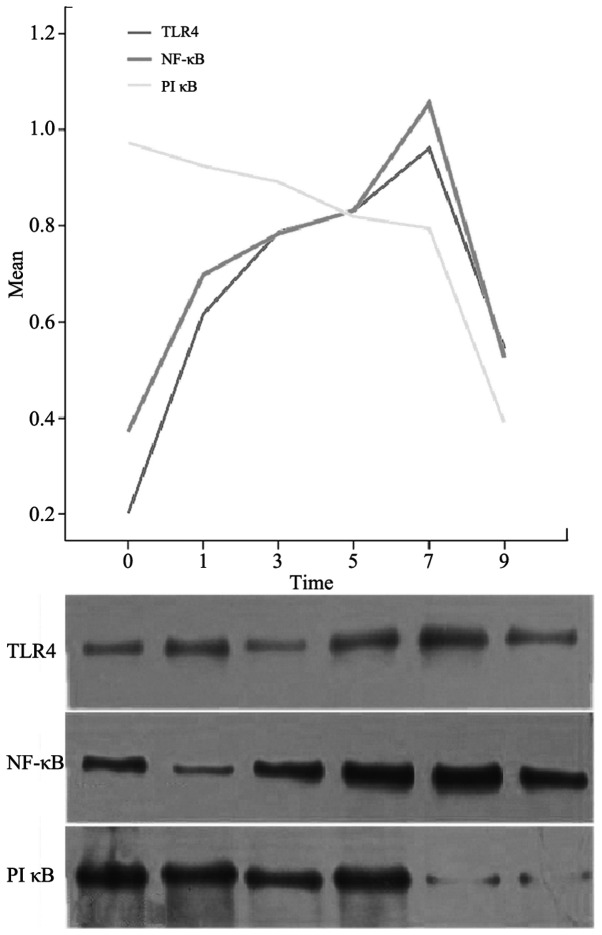
Protein expression of Toll-like receptor (TLR4), NF-κB and PIκB. From day 0 to 1 of high-fat diet stimulation, the expression of TLR4 and NF-κB proteins significantly increased. The protein expression level of TLR4 maintained an increasing trend from day 1 to 7 and the daily rate of increase was less than that on day 1. The protein expression of NF-κB maintained an increasing trend from day 1 to 7 and the rate of increase on days 5–7 was similar to that on day 1. The protein expression levels of TLR4 and NF-κB peaked on day 7 (P<0.05), then demonstrated a decreasing trend. The expression of the PIκB protein demonstrated a decreasing trend from day 0 to 9. The rate of decrease on days 7–9 was significantly different from that on days 0–7, and was the lowest on day 9 (P<0.05). The changing trends of TLR4, NF-κB and PIκB were consistent from day 7 to 9, with no statistically significant difference in rate (P>0.05).

On day 0, no TLR4 or NF-κB protein expression was observed in the mouse intestines; however, PIκB protein expression was present and was at its peak level (P<0.05). At 1, 3, 5, 7 and 9 days, the TLR4 and NF-κB protein expression levels increased progressively until day 7, when peaks were reached (P<0.05). The expression levels then decreased. The expression levels of PIκB protein decreased gradually and reached a minimum on day 9 (P<0.05; Fig. 4).

### RT-PCR

**Figure 5:**
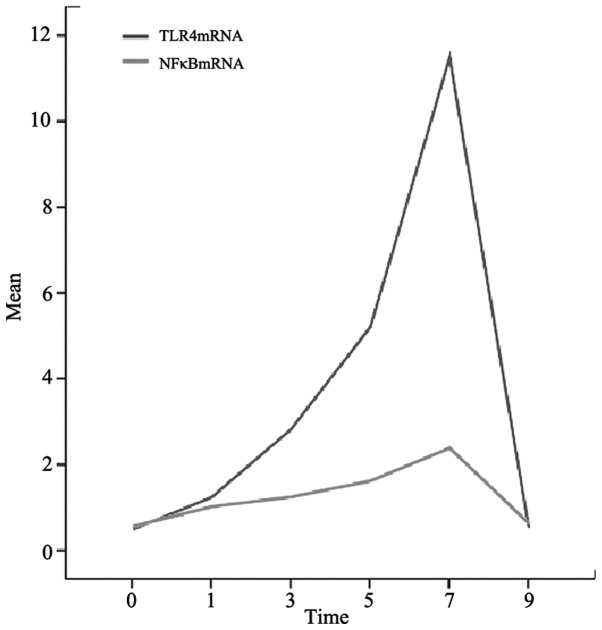
mRNA expression of Toll-like receptor 4 (TLR4) and NF-κB. On day 1 of high-fat diet stimulation, the mRNA expression levels of TLR4 and NF-κB increased; however, the difference between them was not significant (P>0.05). Then, the expression increased significantly, particularly on days 5–7 and the difference in the expression levels of TLR4 and NF-κB reached a maximum. On days 5–7, the change in TLR4 mRNA expression was significantly greater than that over the 1–7-day period (P<0.05). On days 0–5, the change in NF-κB mRNA expression was minor and only a slight increase occurred on days 5–7; however, the increase was not statistically significant (P>0.05). The mRNA expression of TLR4 and NF-κB peaked on day 7 (P<0.05), then began to decline; however, the downtrend trend of the expression of TLR4 mRNA was more marked than that of NF-κB mRNA (P<0.05).

On day 0, there was no expression of TLR4 or NF-κB mRNA in the mouse intestines. On days 1, 3, 5, 7 and 9, the mRNA expression levels of TLR4 and NF-κB increased gradually until day 7, when maximum levels were reached (P<0.05). Then, a gradual reduction was observed. As a reference, β-actin expression remained relatively stable (Fig. 5).

## Discussion

Our results show that a short-term, high-fat diet induces local inflammation in mouse intestine, accompanied by activation of TLR4/NF-κB signaling. This indicates that TLR4/NF-κB signaling is related to the induction of local inflammation in the intestines caused by a short-term, high-fat diet. The results revealed no clear changes in the physiological structure of intestinal tissue following stimulation with a short-term high-fat diet. From day 1 to 9, the intestinal mucosa was complete and a large accumulation of macrophages was not detected. On days 7 and 9, we observed a distribution of macrophages, indicating that the inflammatory reaction shown in this study is different from pathological damage caused by diseases such as ulcerative colitis, since it is low-grade inflammation.

Cani *et al*(16) hypothesized that bacterial LPS acts as a triggering factor, linking inflammation to high-fat diet-induced diabetes and obesity. The authors identified that consumption of a high-fat diet resulted in significant modulation of the dominant bacterial populations within the gut microflora. Additionally, the authors observed a reduction in the number of bifidobacteria, *Eubacterium rectale*-*Clostridium coccoides* species and bacteroides, which favored an increase in the gram-negative to gram-positive ratio. However, in the present study, the observation cycle was shorter than the cycle for bacterial enhancement and LPS release, thus avoiding the interference of LPS generated by intestinal gram-negative bacteria. The results of the present study indicated that a high-fat diet, as an endogenous TLR4 ligand, causes increasing intestinal TLR4 expression. After 1 day of high-fat diet, there was mild activation of intestinal TLR4/NF-κB, which increased gradually, peaking on day 7. According to the analysis of the results, the expression of TLR4 and NF-κB was coincident with the production of TNF-α and IL-6. This is in agreement with the results of the study by Tsujimoto *et al*(17), which demonstrated that the amount of TLR4 expressed was related to the quantity of the inflammatory factor released. PIκB, formed by the phosphorylation of IκB, is the key step for activating the TLR4/NF-κB signaling pathway. With the continual consumption of intracellular PIκB, the activation level of TLR4/NF-κB increased. After 7 days, the expression of TLR4/NF-κB began to decrease. This may signify that PIκB was almost exhausted or a protection mechanism was activated.

The local intestinal inflammatory response and inflammatory factors complement each other. Ou *et al*(18) investigated the expression of TLR4 on human mononuclear/macrophage (THP 1) cell surfaces with flow cytometry. The results demonstrated that the expression of TLR4 on the cell surface may be significantly activated within 24 h after stimulation by IL-6. Abreu *et al*(19) identified that activated NF-κB induces the transcription of TNF-α; TNF-α further promotes the expression of NF-κB. Studies have demonstrated that TNF-α induces the apoptosis of intestinal epithelium cells (20–22), as well as a change in the structure and function of tight junction proteins between cells (23), which leads to increased intestinal epithelium permeability (24) and eventually causes diffusion of the local inflammatory reaction. Inflammatory agents in the local intestinal reaction, including TNF-α and IL-6, may accelerate the activation of the TLR4/NF-κB pathway. This may explain why in the current study the activation of the TLR4/NF-κB pathway demonstrated an increasing trend with time.

Reactive oxygen species (ROS) produced as a result of the activation of TLRs, induce a change in cells. Ko *et al* identified that activation of TLRs in the innate immune response causes retinal photoreceptor oxidative stress and mitochondrial DNA (mtDNA) damage (25). Ye *et al*(26) expanded our knowledge of TLR4 in a well-characterized mouse model of fatty liver disease induced by a westernized diet. The authors identified that a genetic deletion model of TLR4 (Apoe^−/−^/TLR4^−/−^) led to a reduction in high-fat, high-cholesterol diet-induced liver inflammation and injury compared with that observed in wild-type mice (Apoe^−/−^/TLR4^+/+^), which is associated with the reduced expression of ROS and pro-inflammatory cytokines.

The intestine is the body’s largest ‘bank of bacteria and endotoxins’ with a mucous membrane barrier that is highly selective and maintains normal intestinal physiological activities. The intestinal mucous membrane barrier is chiefly composed of mechanical, immunological and biological barriers. The mechanical barrier is primarily constructed of epithelial cells with a tight junction between them; it is the first line of defense against antigens and toxins from outside. TLR4, which is well distributed on the surface, is able to identify pathogens quickly and rapidly induces an immune inflammatory response. The role of macrophages in local intestinal inflammation should not be ignored. The intestinal immune barrier is mainly constructed of secretory immunoglobulin (SIgA) and SIgA is made up of poly-immunoglobulin (pIgA) and pIgA receptors (pIgR). Since there are a number of different types of glycosyls that are significant bacterial ligands on pIgR, the enteric cavity avoids attack by LPS and enjoys immune protection (27). Multiple factors impact the expression of pIgR, including the nutritional state, as well as cytokines, such as TNF-α (28). An increased level of TNF-α induces enterocytes to secrete more pIgR at the protein and molecular level, and to increase intestinal immunoprotection. Furthermore, TNF-α also combines with cell surface receptors, including lymphatic toxin receptors and B-cell activators, to participate in the activation of lymphoid organ genes and enable the transcription of the pIgR gene. This is extremely important in limiting the intestinal inflammation caused by viruses and bacteria and in promoting tissue repair (29). It may explain why the protein and mRNA levels of TLR4 and NF-κB were markedly reduced on day 9 compared with their levels on day 7.

The hypothesis that TLR4 signaling is involved in autoimmune diseases has prompted research into TLR4 inhibition. One study demonstrated that the binding of mAbs to distinct regions on TLR4 inhibits LPS-dependent activation, providing a novel method for manipulating TLR4 activation and also a rationale for designing drugs targeted to TLR4 (30).

As mentioned previously, we successfully established a model of local intestinal inflammation using a high-fat diet. The digested products of the high-fat diet acted as ligands to rapidly activate the intestinal TLR4/NF-κB pathway and cause local inflammation. Whether the local intestinal low-grade inflammation induced by high-fat diet is a prologue to a systemic inflammation response or a partial expression of systemic inflammation remains unknown and requires further study.

## Figures and Tables

**Table I:** Distribution of TNF-α in mouse intestines.

	Time (days)					
	
Score	0	1	3	5	7	9
−	6	5	4	2	0	0
+	0	1	2	4	4	2
++	0	0	0	0	2	2
+++	0	0	0	0	0	2

TNF, tumor necrosis factor.

**Table II:** Distribution of IL-6 in mouse intestines.

	Times (days)					
	
Score	0	1	3	5	7	9
−	6	5	4	2	1	1
+	0	1	2	3	3	2
++	0	0	0	1	2	2
+++	0	0	0	0	0	1
